# Efficacy of alendronic acid solution in removal calcium hydroxide from root canals

**DOI:** 10.4317/jced.61096

**Published:** 2024-05-01

**Authors:** Rebeca Mejía-Haro, María-Verónica Méndez-González, Norma-Verónica Zavala-Alonso, Marine Ortiz-Magdaleno, Mariana Gutierrez-Sánchez

**Affiliations:** 1 Master’s students in endodontics, Endodontics Postgraduate Program, Faculty of Stomatology, Autonomous University of San Luis Potosí, San Luis Potosi, Mexico; 2 Researcher-Professor, Endodontics Postgraduate Program, Faculty of Stomatology, Autonomous University of San Luis Potosí, San Luis Potosi, Mexico; 3 Researcher-Professor, Master of Dental Sciences, Faculty of Stomatology, Autonomous University of San Luis Potosí, San Luis Potosi, Mexico; 4 Specialty in Aesthetic, Cosmetic, Restorative, and Implantological Dentistry, Faculty of Stomatology, Autonomous University of San Luis Potosí, San Luis Potosí, Mexico

## Abstract

**Background:**

Intracanal medication is considered an alternative after instrumentation in the case of pulp necrosis. However, the elimination of this medication plays an important role in the obturation process, which is why various chelating solutions have currently been studied for this purpose. Therefore, this study aimed to analyze the irrigant based on alendronic acid (AA), which contains in its chemical structure functional groups capable of forming complexes with Ca2+ ions.

**Material and Methods:**

90 single-rooted teeth were crowned and standardized to a length of 13 mm. They were instrumented with a progressive K file (Dentsply Maillefer) from #15 to #30. Ca(OH)2 was placed as intracanal medication for 7 days. The roots were randomly divided into the following 4 experimental groups (n=13) according to the irrigant used: 2.25% NaClO, calcium suspension, 0.22% AA, and 10% citric acid; as well as negative (n=5; saline solution) and positive controls (n=5, 17% EDTA). The roots were divided longitudinally and the % of permeable dentinal tubules (% PDT) was determined by thirds (cervical, middle and apical), by analyzing the micrographs obtained by scanning electron microscope (SEM) and the imageJ program. The quantification of the remaining Ca2+ was determined using the Arsenazo III technique.

**Results:**

The Kruskal Wallis test was used for the % of permeable dentinal tubules where a significant difference was determined for the different thirds (*p*<0.005). In the apical third, citric acid and AA irrigants presented a median of 17.71 and 17.51 % PDT respectively. In relation to the quantification of the remaining total calcium, the lowest value was found with AA with a concentration of 4.83 mmol/L.

**Conclusions:**

The 0.22% AA solution has the same capacity to remove Ca(OH)2 from the root canal walls as 17% EDTA and 10% citric acid.

** Key words:**Alendronic acid, calcium hydroxide, citric acid, EDTA, irrigant solution,root canal.

## Introduction

In endodontic treatment, the elimination of persistent microorganisms in the root canal through biomechanical preparation is one of the main objectives to guarantee its success, prior to the filling process. The inflammation of the pulp tissue can be caused by bacteria that are mostly of anaerobic origin, which causes alterations at the pulp level (1,2). When the inflammation process is persistent than the body’s ability to defend itself, an accelerated production of microorganisms occurs in the tooth tissues, destroying the structure of each tissue, and causing the pulp to enter a process of destruction, causing pulp necrosis.

The intracanal medication in the treatment of root canals is considered as an alternative after biomechanical instrumentation, thus reaching places that are difficult to access, such as the branches and isthmuses of the main canal, with Ca(OH)2 which is the most widely used medication for this purpose (2-6). The antimicrobial activity of Ca(OH)2 is associated with its alkaline pH 12.5 that modifies the pH of the microenviroment eliminating remaining microorganisms, inhibit bacterial growth and stimulate periapical repair of the tooth. Properties attributed to the dissociation of the compound into calcium ions (Ca2+) and hydroxyl ions (OH-) that generate damage to the bacterial cytoplasmic membrane, protein denaturation and DNA damage (3,7-10).

However, the total removal of the Ca(OH)2 is a crucial factor in the three-dimensional obturation process, since the remnants of the material in the root canal interfere negatively with the dentin-sealant adhesion interface, reducing the long-term success of endodontic therapy (11-14). Ca(OH)2 remnants affect the physical properties of the sealant, modifying the setting time, decreasing the flow, as well as a physical barrier that obstructs the penetration of the sealant in the dentinal tubules (3-5,15,16). Therefore, Ca(OH)2 must be effectively removed from the root canal walls, as the remnants may be important for the prognosis of treatment (17,18). However, the use of strongly chelating irrigants such as EDTA and NaClO can may cause changes in the hardness and roughness of the dentin, as well as erosion in the walls of the root erosion of the root canal dentin walls, thus causing damage to the dentin walls (18,30). The action of EDTA is not self-limiting, and its chelating effect continues until all the available solution forms complexes between the salt and the calcium present in the hydroxyapatite of the dentin (21,22). And NaClO denatures the collagen on the dentin surface, affecting its elastic modulus and flexural resistance (23,24). In addition to this, currently no irrigating solution has been found that manages to completely eradicate Ca(OH)2 within the root canal (2014).

Alendronic acid (AA) is a chemical compound approved by the Food Drug Administration (FDA), which belongs to the family of bisphosphonates, with hydroxyl and amino surface groups, which give it the property of combining with divalent and trivalent positive ions, to form complexes stable and be used as an irrigating agent (25,26). The objective of this study was to evaluate a new 0.22%, (AA) solution and compare it with different endodontic irrigating solutions (2.25% NaClO, 17% EDTA, calcium slurry, and 10% and citric acid), in the elimination of a Ca(OH)2 paste as an intracanal medication.

## Material and Methods

-Materials

This study followed the ethical recommendations for research in humans and was approved by the Research Ethics Committee of the Faculty of Stomatology, UASLP, with code CEI-FE-030-022. 90 single-rooted teeth recently extracted for periodontal diseases, prosthetic and orthodontic reasons were recollected. Mesiodistal and buccolingual periapical radiographs were obtained to select the tooth with the inclusion criteria: straight single-rooted teeth (degree of curvature less than 5°), fully formed apices, and absence of calcifications or previous endodontic treatment.

The selected teeth were disinfected with 5.25% NaClO and stored in absolute humidity before the experimental tests. The crown was removed from the teeth from the cementoenamel junction with a low-speed handpiece and irrigation with a diamond disc, the roots were standardized to a length of 13 mm. The roots were instrumented using a K-File #10 file (Dentsply Maillefer), inserting it into the canal until the file became visible in the apical foramen and was reduced to 1mm. Subsequently, the root canals were instrumented using the modified lateral technique (27), with fourth progressive files (15, 20, 25 and 30), and irrigating with 2.25% NaClO using an endodontic needle (Endoeze) between each file.

Subsequently, widening of the middle and cervical third was performed with Gates Glidden (GG) low-speed drills, starting with a number 1 drill, until reaching a distance of 2 mm before the working length (11 mm), and was completed by instrumenting with a Gates Glidden #2 bur up to 4 mm before the working length (9 mm). At the end of instrumentation, the roots were stored in saline solution at room temperatura. All procedures were performed by a single operator.

Subsequently, they were subjected to the protocol established by the technique of Haapasalo *et al*.,(17) for the removal of organic and inorganic tissue. For this, the roots were placed in a beaker in an ultrasonic bath (BioSonic UC50) with 50 mL of 5.25% NaClO, followed by 17% EDTA and distilled water between each of them for periods of 4 min. They were enumerated and randomized into each of the groups and sterilized in an autoclave at 121°C for 20 min. Each root was mounted within a system designed to simulate complete isolation by the placement of a rubber dam and clamp. A final irrigation protocol was performed with 2mL of 17% EDTA to permeate the dentinal tubules, followed by 2mL of 70% ethyl alcohol to dehydrate. Root canals were blotted with paper points of the same caliber as the final file. Finally, the roots were medicated with a creamy consistency Ca(OH)2 paste (VIARDEN) and Propylene Glycol, which was placed inside the root canal with a 3mL syringe and Capillary endodontic tip up to the working length. Control radiography was taken to observe the filling of the root canal and were stored for 7 days at 37°C in absolute humidity.

The temporary restoration (CavitTM W (3M, ESPE)) was removed and the Ca(OH)2 paste was initially removed with 3 mL of saline solution and a #30 and #35 file at working length to create a space for the irrigation needle. Finally, was irrigated with 3 mL of the irrigant solutions evaluated: Ggroup A (Saline solution -Negative Control Group, n=5); Group B (EDTA 17%-Positive Control Group, n =5); Group C (2.25% NaClO, n=13); Group D (Calcium slurry, n=13; 14.7g were mixed in 40 mL of saline creating a supersaturated solution of CH); Group E (alendronic acidAA, n = 13; 4 Tablets (130.67 g total weight) of AA with 0.67 g of the active ingredient were dissolved in 130 mL of distilled water and the pH adjusted to 7), and Group F (10% citric acid 10%, n=13).

-Evaluation of the % patent dentinal tubules by SEM

For the experimental groups, Nnine roots were randomly selected and divided into two halves along the coronal-apical axis, and three for each of the control groups. Grooves were first made along the long axis of each root using a diamond disc and a low speed handpiece. Subsequently, the roots were split longitudinally into two halves using a chisel and mallet. Subsequently, the samples were fixed with glutaraldehyde grade I, at 2%, for 24 h and dehydrated by means of an alcohol solution with serial concentrations of 20, 40, 60, 80, 90, 96% until reaching the absolute degree, submerging them. for 10 min at each of the concentrations. At the end of dehydration, the samples were dried and fixed on aluminum pins to be plated with gold (for 60 s at 4 mA) for observation in the scanning electron microscopy on a JEOL JSM-6510 (Hillsboro, OR, USA) at 5kV with 2000x magnification in the different thirds.

**Figure 1:**
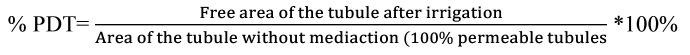
Formula.

Micrographs of the thirds (cervical, middle and apical) of the root canal were obtained with scanning electron microscopy (SEM). The % of permeable dentinal tubules was determined by analyzing the micrographs, which were processed with the Image Thresholding tool of the ImageJ program and using the following expression: (Fig. 1)

% PDT =(Free area of the tubule after irrigation)/(Area of the tubule without medication (100% permeable tubules))*100%

Nine micrographs per third of the root canal walls were processed and analyzed for the determination of the % PDT. A higher percentage of patent dentinal tubules is associated with a greater capacity for Ca(OH)2 removal from the irrigation solution.

-Quantification of Ca2+ using the Calcium Arsenazo procedure

Arsenazo III (Sigma Chemical Co., St. Louis, Mo.), is a chromophore that generates a colored complex with the interaction of Ca2+ ions and the intensity of the chromophore formed is proportional to the total calcium concentration, which can be quantified with a photometer at 650nm (29). Four roots from each experimental group with an average weight of 300 mg were selected and placed in an eppendorf tube with 250 µL of deionized water, and 2 for the control groups. Subsequently, they were subjected to ultrasound (BioSonic UC50) for 1 h. 7.5 µL were taken and seeded in 96-well plates in triplicate with 100 µL of Arsenazo III reagent. Finally, the absorbance was measured in a photometer (Thermo Scientific Multiskan FC). Absorbance data was determined using Thermo Scientific SkanIt™ software.

Where the absorbance was obtained in triplicate for each of the samples analyzed in the different groups.

-Statistic Analysis 

Data were analyzed using statistical software (Sigma Plot, version 12.0, Systat Software Inc., Chicago, IL, USA). The Shapiro Wilk test was performed to verify the normality of the data for the % of permeable dentinal tubules and the quantification of Ca2+ ions. One-way Kruskal-Wallis analysis of range variance and Tukey’s post hoc test was used to identify significant differences in means between study groups. The quantification of Ca2+ ions was analyzed with the t-Bonferroni test for multiple comparisons of the different groups. The significance level was set at 95% for all analyses.

## Results

**Figure 2:**
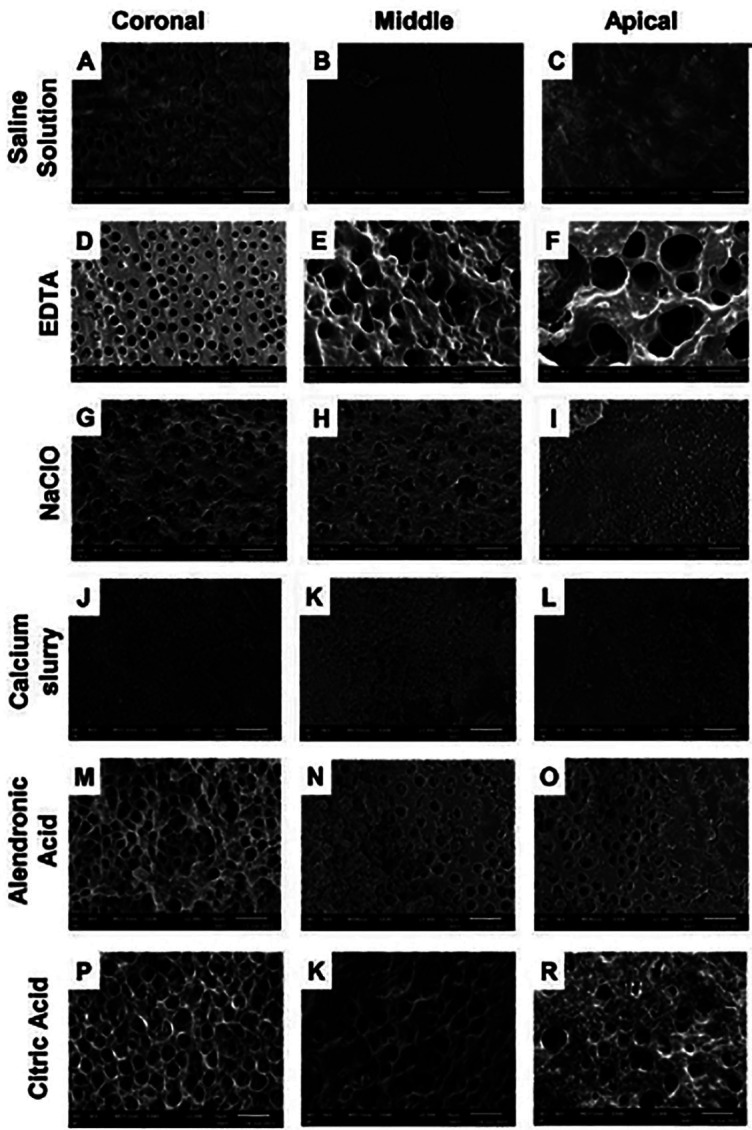
Micrographs of permeable tubules and/or obstructed by the remnant of CH, in the different thirds. Group A (Saline solution); Group B (17% EDTA); Group C (2.25% NaClO); Group D (Calcium slurry); Group E (AA), and Group F (10% Citric acid).

Figure 2 shows the representative micrographs after passive irrigation with each of the irrigating solutions in the different thirds. In them, it can be observed that the walls of the root canal in the different thirds are totally obstructed for the samples of Group A and D corresponding to the Saline Solution and Calcium Milk respectively. Likewise, it can be seen that the samples irrigated with NaClO partially permeate the dentinal tubules in the middle and coronal third. While the apical third is totally obstructed. In the case of EDTA, citric acid and AA chelating solutions, a greater number of dentinal tubules are observed, especially in the cervical third, including slight erosions in the dentinal tubules. Effect that can be more easily appreciated in the samples irrigated with EDTA and citric acid (Fig. 2 E,F,K).

**Figure 3:**
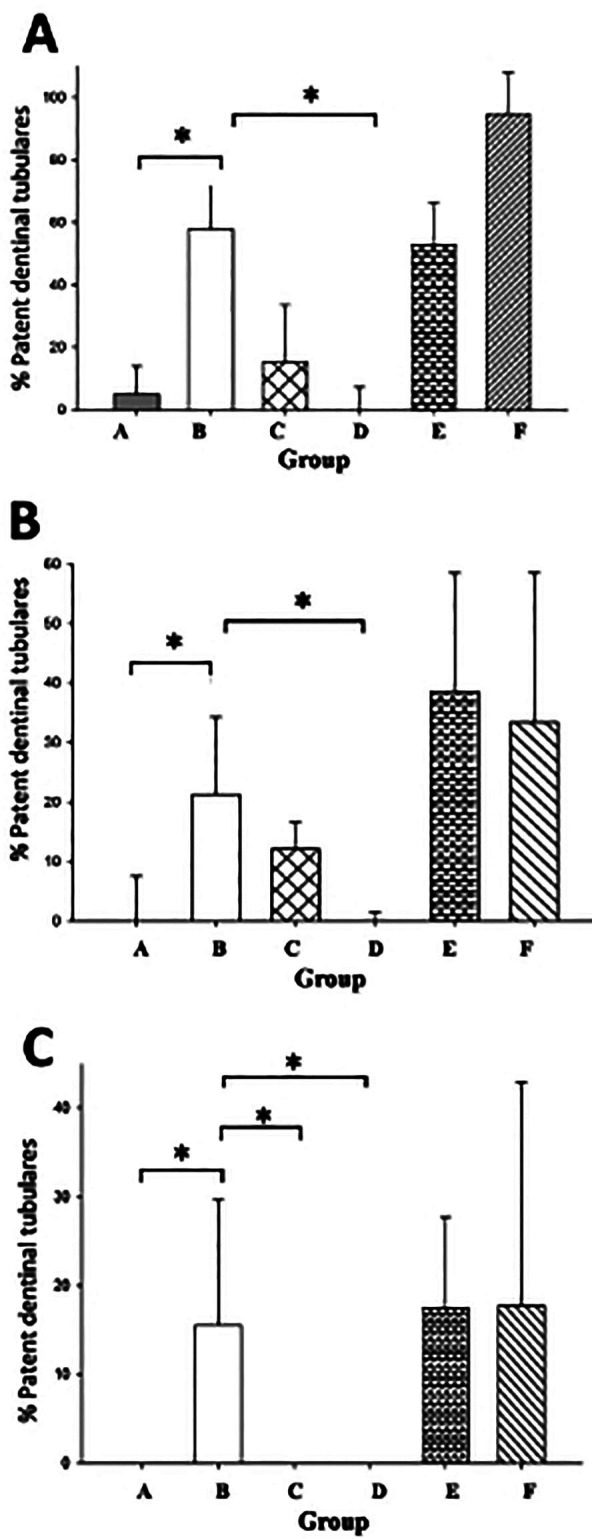
% Patent dentinal tubules in the three different thirds, determined using the program in imageJ. (A) Coronal third, (B) Middle third and (C) Apical third. Group A (Saline solution); Group B (17% EDTA); Group C (2.25% NaClO); Group D (Calcium slurry); Group E (AA), and Group F (10% Citric acid).

In Figure 3 the results of the % PDT to different thirds are presented. It can be seen that the highest values of %TFD were obtained for the chelating solutions. In other words, the highest rate of Ca(OH)2 removal was obtained with the irrigating solutions based on EDTA (57.83%), citric acid (94.56%) and AA (52.91%) for the coronal third; EDTA (21.23%), citric acid (33.45%) and AA (38.58%) for the middle third; EDTA (15.57%), citric acid (17.71%) and AA (17.52%) for the apical third.To according to the statistical analysis, no significant difference was found for these groups in the different thirds (*p*=<0.001).

**Figure 4:**
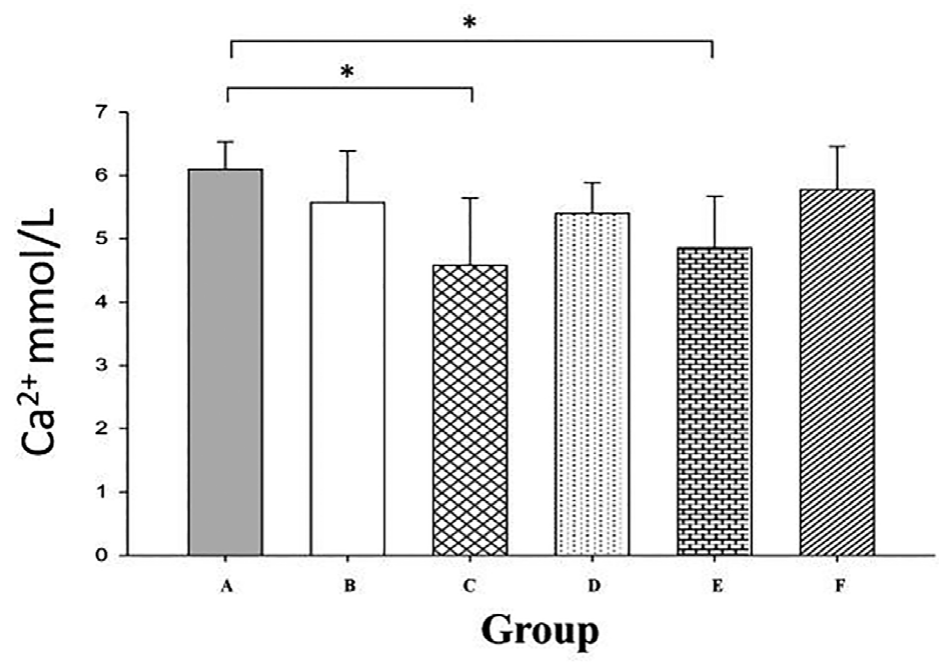
Quantification of Ca2+ using the Calcium Arsenazo procedure in the root canal after irrigation. Group A (Saline solution); Group B (17% EDTA); Group C (2.25% NaClO); Group D (Calcium slurry); Group E (AA), and Group F (10% Citric acid).

Calcium in a neutral medium forms a blue complex with Arsenazo III (1,8-dihydroxy-3,6-disulfo-2,7-naphthalene-bis(azo)-dibenzenarsonic acid). And according to Beer Lambert’s law, the intensity of the color is directly proportional to the amount of calcium in the solution. And based on this, the amount of Ca2+ remaining in the root canal associated with the medication in the root canal was determined by spectrometry using the Calcium Arsenazo procedure. Figure 4 shows the meanaverage and standard deviation of the amount of Ca2+ ions remaining in the root canal obtained by the arsenazo technique.

The mean and the standard deviation obtained from each of the irrigating solutions can be seen in Figure 4. The Shapiro-Wilk statistical test was performed to check the normality of the set of results obtained from the absorbance test. And the *p*=0.012 value of the tests indicates that the data do not follow a normal distribution. Therefore, the t-Bonferroni test was performed for multiple comparisons of the different groups. And the results showed a significant difference in the Sodium Hypochlorite Group (4.543±1.097mmol/L) and AA (4.843±0.828mmol/L) with respect to the negative control (6.081±0.473mmol/).

## Discussion

It should be remembered that drug residues affect the adhesion of the sealing materials and are critical in the coronal third in terms of the restorative process, since at this point, the sealants are under occlusal forces, as well as condensation and pressure from the teeth, sealing materials and post placement (19,20). In the present study, carried out on single root extracted human teeth, it was used as an experimental model to evaluate the efficacy of a new AA irrigating solution versus conventional irrigating agents, using two quantitative methods such as the % patent dentinal tubules obtained from the analysis of micrographs obtained by SEM using ImageJ software, and quantification of remaining calcium using the calcium-arsenazo assay.

The use of tools such as the ImageJ program provides us with greater precision during the determination of the % of permeable dentinal tubules from the micrographs obtained by SEM. Which are processed and standardized to a scale that more accurately isolates the areas to be analyzed. In our results, we show that EDTA is an excellent chemical chelator for root canal treatment, and that it has been evaluated as the best for smear layer removal in multiple studies due to its four acetate groups and two amino acids. However, prolonged exposure to strong chelants, such as EDTA or citric acid, can weaken root dentin (21-26). EDTA can have clinical implications, since it causes excess decalcification of the dentin of the canal, generating erosion of the dentinal tubules and affecting their physicochemical properties. The action of EDTA is not self-limiting, that is, its chelating effect continues until all the available solution forms complexes between the salt and the calcium present in the hydroxyapatite of the dentin. The results of this study show that it is possible to consider AA as a chelation solution in clinical practice. AA is aAnd it is that this chemical compound that belongs to the family of bisphosphonates approved by the FDA with superficial hydroxyl and amino groups, and that even at the low concentration evaluated (0.22%), has an important chelating capacity that is appreciated in the micrographs to the different thirds without erosion of the dentinal tubules.

Several studies have compared irrigation solutions based on chemical compounds of the bisphosphonatose family for this purpose, such as the work carried out by Sherin J. *et al*., in their study the efficiency of irrigation protocols for the removal of Ca(OH)2 (Group I: 17% EDTA; Group II: 10% citric acid; and Group III: 18% etidronate). Where Group III (coronal third) and Groups I and II (middle third) acquired the highest cleaning pressures; Groups II and III (apical third) presented the lowest pressures. Comparing the thirds, all the groups showed differences in the proportions. The pH of Groups I, II and III was 6.8, 1.4 and 0.3 respectively. In this investigation he concluded that the highly alkaline pH of Ca(OH)2 increases the pH of citric acid towards neutrality. where it becomes an ineffective chelator; on the contrary, the high acidity of etidronate compensates for its weaker chelation (32). Likewise, recent studies by Deari S. *et al*., compared EDTA and HEDP (1-hydroxyethane-1.1-diphosphonic acid) solutions with respect to their ability to solubilize calcium from dentin. They prepared solutions with a molarity corresponding to that of 17% EDTA by adjusting their pH to 8.5 by dissolving HEDP and sodium hydroxide salts in deionized water. Discs of previously standardized radicular dentin were prepared and submerged for 1 min. Dissolved Ca2+ is prolonged by atomic absorption spectroscopy, and dentinal tubules appear apparently permeable by laser scanning microscopy and automated image analysis. Solutions prepared from tetrasodium salts were alkaline (pH 11.3–11.4). while the counterparts made from disodium salts were acidic. EDTA solutions dissolved more calcium than HEDP counterparts. And there was a high connection between the dissolved calcium and the apparently open tubular areas. The differences between groups regarding open tubules were similar to those observed regarding Ca2+ values (33). Likewise, these results support the research carried out by Chen Goldenberg *et al*., who compared the cleaning and erosion of the root canal walls after the use of an irrigant based on HEDP with the conventional irrigation of sodium hypochlorite followed of EDTA, both applied passively (34). In both groups there were more cases with smear layer in the apical third of the root canal than in the coronal third. but the groups did not differ significantly from each other. The walls in both groups were almost free of debris. However, moderate root dentin erosion was found in 10-26% of cases in both groups, but severe erosion was detected in only one case in the EDTA-irrigated group, which does not show a significant difference between the groups. However, part of the limitations of the AA solution is the low solubility of AA in aqueous medium (0.22%). As well as the fact that it is an *in vitro* test, where the specimen lacks intrinsic factors such as pH, temperature and humidity. However, the perspectives include evaluating the physicochemical properties of dentin after exposure to the AA solution prior to an *in vivo* test. Since the micrographs show a slight erosion in the dentinal tubules in the group irrigated with AA, and an elimination of Ca(OH)2 similar to that of EDTA and citric acid.

## Conclusions

The present study demonstrated that the manual irrigation protocol is not efficient in the removal of the root canal walls, especially in the apical third and regardless of the nature of the irrigant. However, AA showed similar results to EDTA solutions and citric acid despite being in a low concentration of 0.22%, so it could be recommended for the removal of HC from root canals without erosions in the dentinal tubules.

## Data Availability

The datasets used and/or analyzed during the current study are available from the corresponding author.
